# Thiamine deficiency unrelated to alcohol consumption presented with urinary retention and Wernicke's encephalopathy: A case report

**DOI:** 10.1002/ccr3.7681

**Published:** 2023-08-10

**Authors:** Takafumi Uchi, Shingo Konno, Hideo Kihara, Hideki Sugimoto

**Affiliations:** ^1^ Department of Neurology Toho University Ohashi Medical Center Meguro‐ku Japan

**Keywords:** neurological symptoms, reflux esophagitis, thiamine deficiency, urinary retention, vitamin supplementation

## Abstract

Thiamine deficiency can present with rare neurological symptoms such as urinary retention, along with common symptoms like ataxia and decreased limb muscle strength. Early recognition and treatment are crucial to improve symptoms and prevent complications.

## 1 INTRODUCTION

Thiamine deficiency is characterized by a broad range of neurological signs, including impaired reflexes, symmetrical motor, and sensory deficits in the extremities, myelin loss without acute inflammation, and Wernicke encephalopathy.^1^ Early signs of thiamine deficiency include peripheral neuropathies in adults and adolescents as well as infant fussiness and irritability.^2^ Additionally, thiamine deficiency can cause neurological symptoms such as anorexia, agitation, muscle pain, and gait disturbances; oculomotor signs such as spontaneous gaze nystagmus; and disturbed eye tracking.^3^, ^4^ This article describes a case of thiamine deficiency with rare symptoms, including orthostatic hypotension and urinary retention, accompanied by common neurological symptoms.

## 2 CASE HISTORY

**FIGURE 1:**
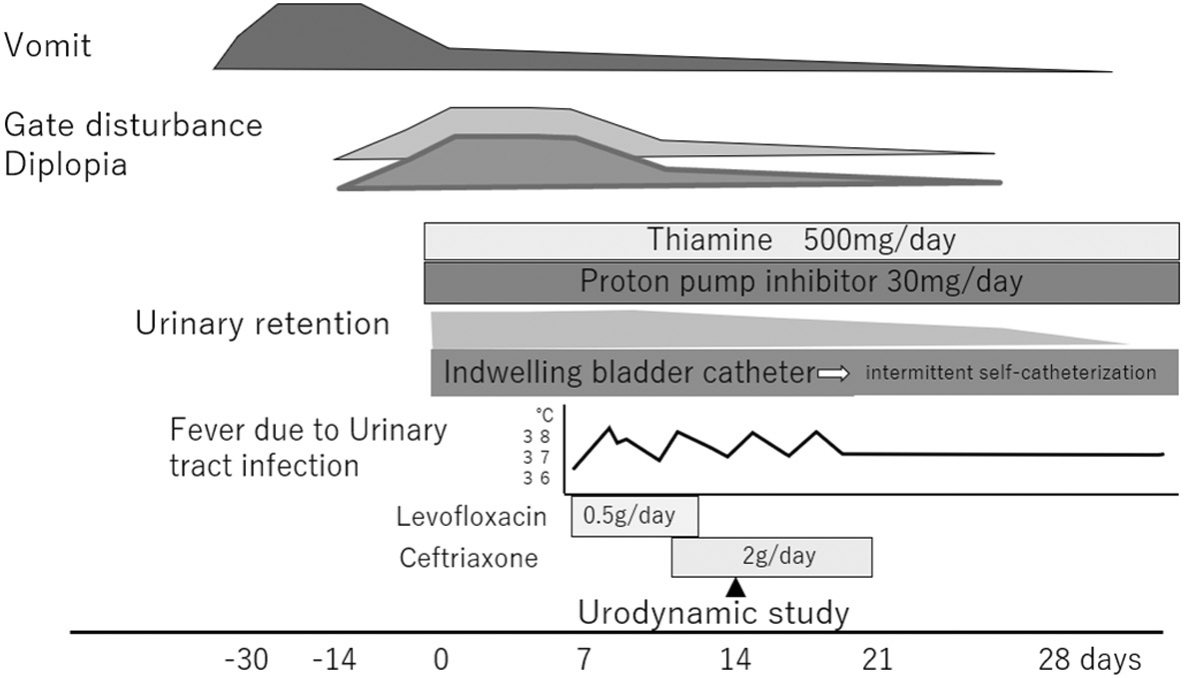
Patient's clinical course.

Figure 1 shows the clinical course of the patient. A 65‐year‐old male patient presented to the emergency room with gait disturbances after experiencing repeated episodes of vomiting and appetite loss, starting 1 month before admission. The patient had a history of reflux esophagitis and had been taking proton pump inhibitors (PPIs); however, he had discontinued taking PPIs on his judgment. The patient was not a habitual drinker and had no history of diabetes, or preexisting dementia, including Alzheimer's disease.

### 2.1 Differential diagnosis, investigations, and treatment

**FIGURE 2:**
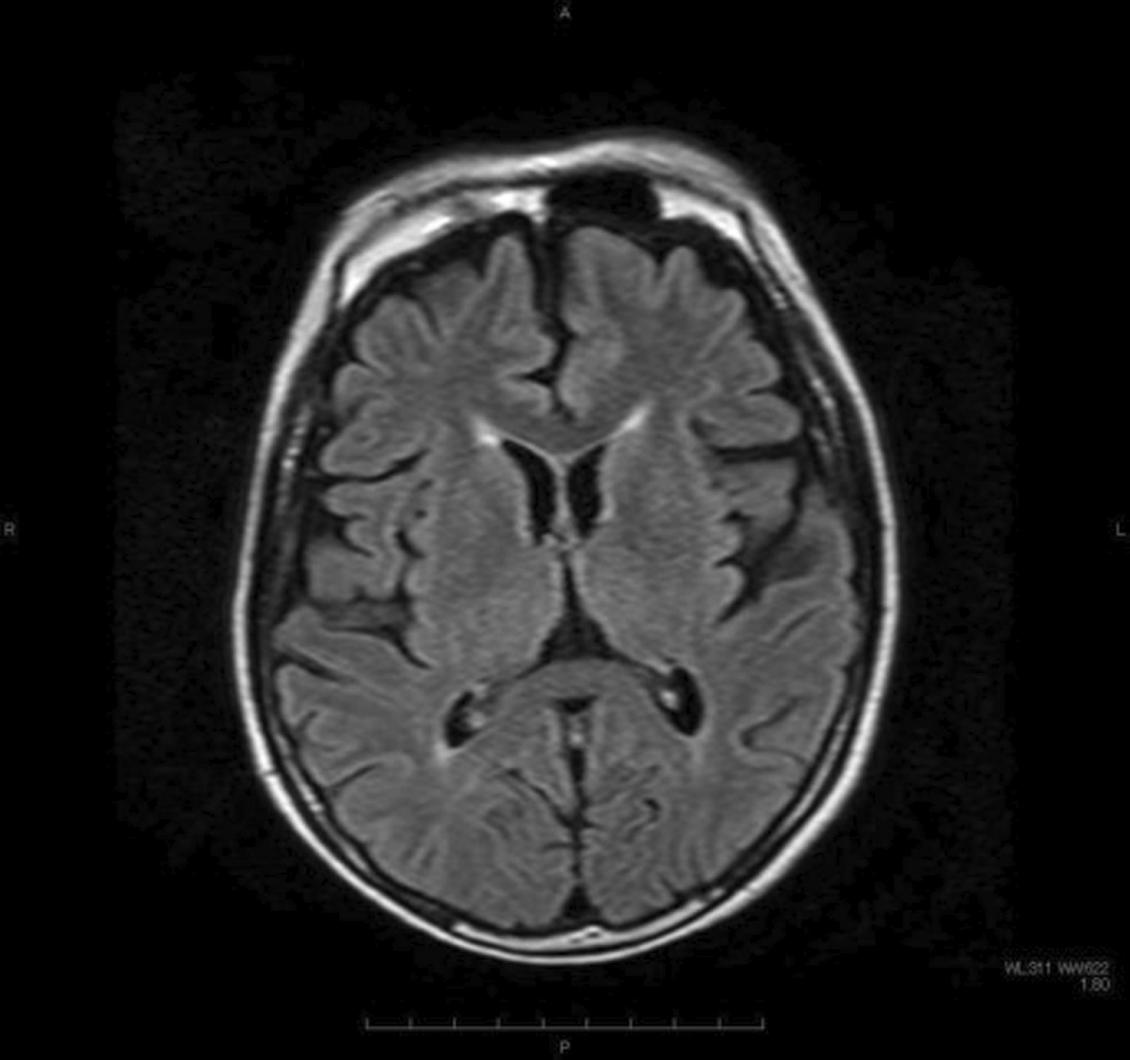
Brain magnetic resonance imaging findings. Brain magnetic resonance imaging on admission showed brain atrophy, but no abnormal findings in thalamus.

**FIGURE 3:**
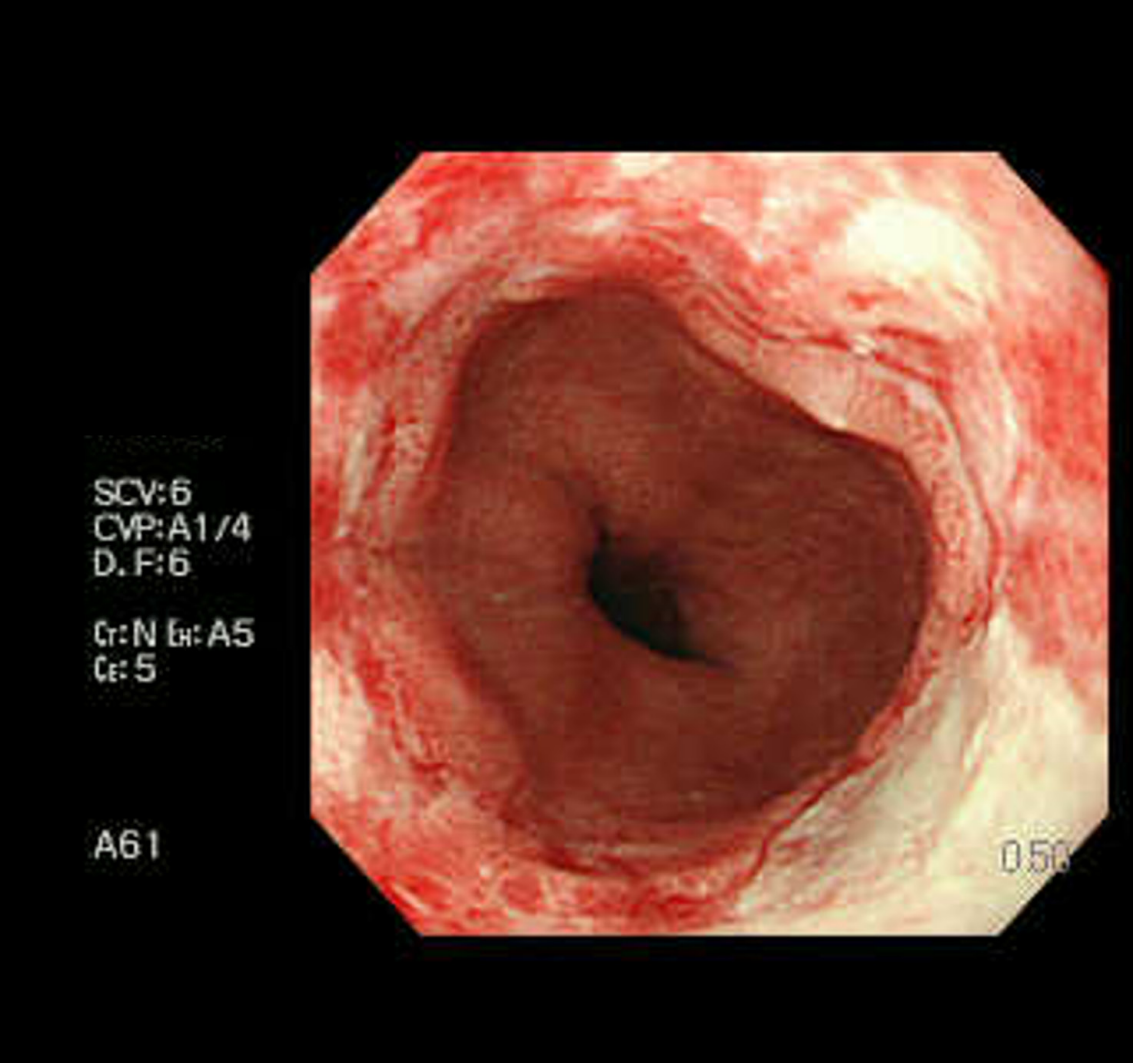
Gastrointestinal fiberscope findings. Gastrointestinal fiberscope revealed hiatal hernia and Grade D of reflux esophagitis.

On physical examination on admission (Day 0), consciousness was classified as E2V5M4 based on the Glasgow Coma Scale. Eye movements were fixed in the middle of both eyes, limb muscle strength was four out of five on Manual Muscle Testing with no between‐side difference. Tendon reflexes were decreased in all four limbs; moreover, no pathological reflexes were detected. The patient could not independently walk due to ataxia. The patient had an indwelling bladder catheter due to urinary retention. A magnetic resonance imaging head scan performed on Day 0 revealed mild cerebral atrophy; however, he lacked abnormal signs in the thalamus (Figure 2). On Day 2, a gastrointestinal scope revealed severe reflux esophagitis (Figure 3). The peripheral nerve conduction velocity testing revealed a pattern of axonal damage in both motor and sensory nerves in the extremities. The coefficient of variation of the R‐R interval on electrocardiography was 1.8% (>2.8%).

Daily intravenous infusions of 500 mg thiamine and PPIs were started immediately after the visit (Day 0). Vitamin B2, B12, and folic acid levels were within the normal range, except for serum thiamine (<20 ng/mL) which was taken prior to administration of thiamine. Prostate‐specific antigen levels were normal. Diplopia and gait disturbance showed relatively early improvement from Day 7 and disappeared until Day 28; however, dysuria persisted and he had recurrent fevers due to urinary tract infectious disease. He received 500 mg/day of levofloxacin or 2 g/day of ceftriaxone on each occasion.

### 2.2 Outcome and follow‐up

A urodynamic study revealed insufficient bladder contraction during voiding attempts, detrusor underactivity, and residual urine volume >30 mL. After antibiotic administration and intermittent clean self‐catheterization at intervals of 4–5 h, the fever disappeared and the patient was discharged on Day 35.

## 3 DISCUSSION

Thiamine deficiency is prevalent and underdiagnosed among acutely and chronically ill individuals in high‐income countries.^5^ Although alcoholism is an established cause of thiamine deficiency due to poor eating habits, it is not the only cause.^6^ Nonalcoholic causes of thiamine deficiency include malabsorption syndromes, bariatric surgery, chronic diarrhea, prolonged fasting, and hyperemesis gravidarum.^1^, ^2^ In the present case, the main causes of thiamine deficiency were vomiting and anorexia due to gastroesophageal reflux. Chronic thiamine deficiency can result in vomiting, constipation, diarrhea, and anorexia due to acute thiamine deficiency‐related illnesses.^2^ Accordingly, thiamine deficiency may have worsened the abdominal symptoms in this patient.

Even mild‐to‐moderate vitamin deficiencies can cause functional changes in the autonomic nervous system.^7^ Although peripheral neuropathies secondary to vitamin deficiencies such as thiamine deficiency can be difficult to diagnose, they are frequently encountered.^8^

Thiamine was the first vitamin to be identified as a catalyst for energy generation through decarboxylation.^1^ A review of thiamine deficiency and related neurological and psychiatric disorders showed that thiamine deficiency can cause various neurological symptoms, including dysautonomia.^3^ Additionally, individuals with postural orthostatic tachycardia syndrome, which is a type of dysautonomia, have been shown to have vitamin B12 and thiamine deficiency.^9^ Although these studies provide some evidence regarding the relationship between thiamine deficiency and autonomic nerve failure, further studies are warranted to determine the extent of this relationship and whether other factors contribute to dysautonomia.

In summary, thiamine deficiency leads to autonomic dysfunction. Prophylactic medical interventions for infection may be effective against urinary dysfunction, which may persist as a sequela even after initiation of thiamine supplementation.

## AUTHOR CONTRIBUTIONS

**Takafumi Uchi:** Writing – original draft. **Shingo Konno:** Writing – original draft; writing – review and editing. **Hideo Kihara:** Supervision. **Hideki Sugimoto:** Supervision.

## FUNDING INFORMATION

No funding received.

## CONFLICT OF INTEREST STATEMENT

The authors declare that they have no conflict of interests.

## ETHICS STATEMENT

This study was conducted in accordance with the guidelines of Toho University and did not require ethical approval.

## CONSENT

Written informed consent was obtained from the patient to publish this report in accordance with the journal's patient consent policy.

## Data Availability

The data sets supporting the findings of this study are available from the corresponding author upon reasonable request.
